# An Examination of Community Awareness and Understanding of Patron Banning Provisions in Western Australia: Implications for Policy Development and Success

**DOI:** 10.1007/s10610-022-09531-9

**Published:** 2022-10-10

**Authors:** Clare Farmer, Peter Miller, Sally Kennedy, Jessica Saligari, Emma Gretgrix

**Affiliations:** 1 School of Humanities & Social Sciences, Deakin University, Pigdons Road, Waurn Ponds, Geelong, VIC Australia; 2 School of Psychology, Deakin University, Geelong, Australia

**Keywords:** Patron banning, Alcohol-related violence, Deterrence, Banning policy

## Abstract

Individuals who engage in problematic behaviours within Australian night-time entertainment precincts can be banned from entering certain locations. Bans are expected to deter recipients and the wider community from further inappropriate behaviours. The collective effect is intended to reduce crime and increase safety within entertainment precincts. This study examined public awareness and understanding of two patron banning mechanisms (police barring notices and prohibition orders) used across Western Australia (WA). An anonymous survey was completed by 1018 respondents, and interviews were conducted with 54 stakeholders. Survey participants had limited awareness of patron banning: 75% had not heard of police barring notices; 87% had not heard of prohibition orders. Knowledge was higher for individuals directly associated with a ban recipient. Stakeholders also perceived a low level of community awareness and understanding of patron banning. Patron banning may have some merit as a specific deterrent for recipients but, in WA, the lack of public knowledge means that the banning provisions may currently have limited effect as a general deterrent. Public awareness should be increased in order to optimise the direct and consequential effects of patron banning policy.

## Introduction 

Various forms of spatial exclusion, prohibition and patron banning are used across Australian and international jurisdictions in response to alcohol-related problematic behaviours, particularly in and around licensed premises — venues where alcohol can be purchased and/or consumed, such as bars, clubs etc. (Farmer, Curtis & Miller 2018; Farmer & Clifford 2021; Johnstone 2017). Powers to prohibit and exclude are presumed to increase community safety by removing troublesome individuals, preventing crime and acting as a deterrent to future undesirable behaviours (Farmer 2018; Miller, Curtis & Palmer 2016). The discourse surrounding the introduction and expansion of patron banning powers presumes a dual deterrent effect (Farmer, Curtis & Miller 2018). Firstly, that the fact of being excluded and the restriction of the right to move freely will deter the recipient from further behaviours which may lead to another ban — particularly where a subsequent ban may be more onerous. Secondly, that the risk of exclusion will deter the wider community from engaging in behaviours which may lead to a ban. Both presumed deterrent effects may be reasonable, but specific research and analysis is limited, particularly with respect to broader community deterrent effects.

A key rationale for the implementation of patron banning across Australia is that their deterrent effect will reduce crime and disorderly behaviour (Farmer, Curtis & Miller 2018). For a patron ban to act as an effective deterrent requires sufficient awareness of the presence of the provisions, and their possible application and consequence for recipients. Recent studies in the Australian state of Victoria examined the experiences and perceptions of licensee barring order recipients (Farmer 2019, 2021a, b), and the offending histories of police ban recipients (Curtis et al. 2022). Both studies focused on the specific deterrent effects of the banning provisions for recipients. Other than a preliminary analysis by Curtis et al. (2018), which used a short online survey to assess the level of awareness of banning provisions in Victoria, the level of community awareness of the various banning mechanisms within each Australian jurisdiction is not known, neither is the extent to which the existence and operation of banning provisions may affect the behaviours of the general population. As a first step to exploring this gap in the understanding of the wider effects of patron banning, this paper examines the awareness and understanding of two provisions currently operational in the state of Western Australia (WA): police barring notices and prohibition orders. The paper first provides an overview of comparable measures addressing anti-social behaviours in other jurisdictions, and associated research examining their deterrent effects. This is followed by consideration of patron banning in Australia and the specific provisions currently used in WA, which form the basis of the analysis in this paper. After setting out the research method, key findings are presented and then discussed in relation to policy expectations of deterrence. Implications for the possible refinement of patron banning policy in WA are set out in the final part of the paper.

## Research Context

### Anti-social Behaviour and Deterrence

The expectation that exclusion from a licensed venue or wider public area can change behaviours and reduce crime presumes that the known risk of being excluded can or will deter future potential anti-social, disorderly and/or violent behaviour. This draws upon a presumption of rational decision making and perceptual deterrence, whereby individuals weigh up the possible benefits of a given act or behaviour against the probable risks (Becker 1968; Mann et al. 2016). A penalty that is framed as a deterrent (such as a patron ban) should have the capacity to influence an individual decision to behave in a particular way, for which awareness of the penalty is a pre-requisite. Such penalties are expected to have a specific deterrent effect on those who have already been subject to them. Their existence and the knowledge of their potential effect is also presumed to act as a general deterrent to unacceptable behaviours across the wider community. Individuals are influenced by a complex blend of personal, social, situational, contextual, psychological, attitudinal and risk-related factors, which can be further complicated by the consumption of alcohol (Taylor, Keatley & Clarke 2020). Measures to address problematic behaviours in and around entertainment districts and specific licensed premises include surveillance and enforcement mechanisms, such as the proximity of police officers, visible CCTV or the use of ID scanners to identify patrons as they enter individual venues. Each has the potential to influence behaviours, but the precise deterrent effects are variable and far from conclusive (Ariel, Bland & Sutherland 2017; De Andrade et al. 2020; 2021; Piza et al. 2019). Perceptions of and responses to risks are also individualised, with some people more likely to be deterred than others (Jacobs 2010; Matthews & Agnew 2008; McGrath 2009).

Deterrence can apply to individuals receiving a particular penalty, more generally to the community through the perceived risk of a sanction being imposed and enforced, or it can reflect more intricate intersections of specific and general effects. The confounding effects of alcohol or drug consumption upon cognisance, rational thinking, risk informed decision making, anti-social and other disorderly behaviours further complicate the paradigm of perceptual deterrence and deterrability. Nightlife crime typically involves impulsivity, sometimes in response to provocation, and almost always when those involved are intoxicated (Fleming 2008; Graham and Homel 2008; Hadfield, Lister and Traynor 2009; Hughes et al. 2008; Mawby 2017; McNamara and Quilter 2015; Miller et al. 2015). The premise of deterrence which provides a core rationale for patron banning arguably denies or ignores fundamental determinants of most nightlife crime and undermines a key source of revenue for the venues from which individuals are then removed.

Mechanisms that exclude recipients from public or private domains operate across a range of international contexts, generally in response to lower-level anti-social and/or disorderly behaviours. They are typically presumed to influence the future undesirable behaviours of recipients and of the community more broadly (Ashworth & Zedner 2008). A body of research has drawn attention to potential criminogenic consequences of lower-level prohibitions and penalties, of which patron bans are an example. The actual deterrent effects are far from conclusive, and there has been limited research examining the associations between general awareness of interventions and the level or extent of general deterrent effects.

Motz et al. (2020) reported the findings of a longitudinal study of British twins which examined the effects, including deterrence, of different types of intervention/contact with the criminal justice system: such as receipt of an anti-social behaviour order (ASBO). ASBOs, introduced in Britain under the *Crime and Disorder Act 1998*, were a civil order with criminal breach provisions. An ASBO could be given to any person aged 10 or older — just under half were imposed on under 18s (Crawford 2009a, b p.7) — found to have behaved in an anti-social manner. ASBOs were intended to deter future anti-social behaviour by imposing certain prohibitions (e.g. locational or association), and identifying the recipient as anti-social (Burney 2002, 2009). Motz et al. (2020) found limited specific deterrent effects from ASBOs, with receipt more likely to be a predictor of future misbehaviour. General deterrence was not addressed in this study.

Under the *Anti-Social Behaviour Act 2003* (ss.30–36), police in England and Wales were afforded powers to disperse groups of two or more people from designated areas where there is believed to be significant and persistent anti-social behaviour. Dispersal powers are akin to anti-loitering laws in the USA which, since the 1980s, have set out to pre-emptively target individuals and groups regarded as problematic but which do not mandate commission of a criminal act to require compliance (Divringi 2014; Palomo 2002). Dispersal orders have the capacity to be applied even more broadly, although they are time limited. The use and potential effects of dispersal orders have been subject to considerable analysis, most notably by Crawford (2008, 2009a, b) and Crawford and Lister (2007). Reflecting upon the requirement for each dispersal order to be clearly publicised, Crawford and Lister (2007) position effective communication as essential to the success of the provisions. The community needs to know about a dispersal order to enable compliance and facilitate its enforcement. Despite the embedded deterrent and preventative expectations of dispersal orders, there has been little research to examine community awareness and understanding.

In the US city of Seattle, Beckett and Herbert (2010) found that police officer powers to issue on-the-spot exclusion orders from public spaces, lasting for up to a year, had few demonstrable beneficial effects upon the behaviour of recipients or that of the general population. Their study did not specifically address public awareness or general deterrence. In a study of responses to police zonal banning powers in Denmark, Sogaard (2018) emphasises how the provisions are presumed to have deterrent and crime reduction effects, and highlights the importance of assessing the success of policy goals, such as the presumed deterrent effects. In challenging the expectation of rational decision-making on the part of patrons, whether banned or not, Sogaard (2018) focuses on specific deterrence (referred to as a linear model of causality and deterrence). The study does not examine general awareness of zonal banning or situate consideration of the success of the provisions against the level of knowledge or understanding. The use of exclusion from public areas in response to anti-social behaviours has also been examined in other countries, such as Germany (Belina 2007), Canada (Sylvestre, Bernie & Bellot 2015), Hungary (Podoletz 2016) and Denmark (Sogaard, Houborg & Pedersen 2017), but without clear evidence of their general deterrent or other broader effect/s upon behaviour.

The expansion of CCTV, framed as a situational crime prevention tool which increases the perceived risk of offending (Clarke 1995), has received more analysis than prohibitive and exclusionary provisions with respect to general awareness. The use of CCTV is not typically individualised: CCTV covers everybody in the proximity of cameras regardless of likely or actual behaviour. Much of a large body of international research examining the impact of CCTV reveals ambiguous results with respect to crime reduction (see, for example, Lett, Hier & Walby 2012; Piza 2018; Welsh & Farrington 2002). Piza et al. (2019) undertook a systematic review of research examining the effects of CCTV on crime. In setting the context for the study the authors acknowledge the link between publicity and improved community awareness as potential ways in which CCTV can contribute to reductions in crime. However, this is not explored in depth within the review. Gill and Spriggs (2005) did address public awareness of CCTV more specifically, in a series of public attitude surveys conducted across the UK prior to and after the installation of CCTV systems. The report identified a high level of public awareness of the cameras in most localities. There was less awareness in large city centre areas where, the authors speculate, the cameras were less apparent within the large amounts of street furniture already present (Gill & Spriggs 2005 p.45). The link between general public awareness and the wider deterrent effect of CCTV was not assessed specifically, but of relevance here is the finding that generally pre-organised offences, such as burglary, vehicle crime, criminal damage and theft were more likely to decrease where CCTV was installed while more spontaneous offences, such as violence against the person and public order offences, did not (Gill & Spriggs 2005 p.59).

Overall, very little research has examined the general deterrent effects of policies and provisions which address lower-level anti-social and disorderly behaviours, despite the embedded assumptions and expectations within the justifications for such provisions. For a provision to have a general deterrent effect, sufficient awareness of the provisions should be evident. Therefore, this paper examines the level of awareness and understanding of two patron banning provisions in WA.

### Patron Banning in Australia

In addition to measures such as tighter licensing regulation, the introduction of last-drinks provisions and networked ID scanners (Miller et al. 2014, 2019), a feature of responses to issues of alcohol-related disorder within entertainment districts across Australia has been the introduction of one or more methods of patron banning (Farmer, Curtis & Miller 2018, Taylor et al. 2018; Farmer & Clifford 2021; Farmer, Clifford & Miller 2021). Three broad types of patron banning are currently in operation in Australia: venue specific bans, typically issued by licensees; police-imposed bans, which generally cover one or more licensed venue and/or public area within entertainment districts; and court-imposed exclusion orders, implemented as part of a more formal outcome, usually with an offender specific prohibitive scope.

Since the mid-2000s, the scope of banning provisions has expanded both legislatively and operationally. Many banning measures can be implemented on-the-spot, and some concern has been expressed about the particular consequences for due process and the individual rights of recipients, as well as a lack of scrutiny of banning as a mechanism to address alcohol-related disorderly behaviours (Curtis et al. 2018; Farmer 2014, 2018, 2019, 2021a, b; Farmer, Clifford & Miller 2020; Miller, Curtis & Palmer 2016; Palmer & Warren 2014; Taylor et al. 2018). Despite limited evidence-based analysis of their effects, all Australian jurisdictions have introduced one or more forms of patron banning. Table 1 summarises the use of the three key types of patron ban in each Australian jurisdiction.

**Table 1:** The use of each type of patron banning across Australian jurisdictions @ 1 July 2021

Ban type	ACT	NSW	NT	QLD	SA	TAS	VIC	WA
Licensee bans	Y	Y	Y	Y	Y	Y	Y	Y
Police-imposed ban	N	Y	Y^1^	Y	Y	Y	Y	Y
Court-imposed exclusion order	Y	Y^2^	Y	Y	Y^4^	Y^4^	Y	Y^3, 4^

^1^Alcohol Protection Orders: imposed by police, also include provision for a ban from licensed premises

^2^Place Restriction Orders or Area Restriction Orders: available to the courts; not specific to alcohol-related issues, can be used in response to serious alcohol-related offending

^3^Prohibited Behaviour Orders: not specific to alcohol-related issues, but can exclude recipients from licensed premises and entertainment districts

^4^Prohibition Orders: imposed centrally by the Department of Racing, Gaming & Liquor rather than via the courts, not specific to alcohol-related issues but can exclude recipients from licensed premises and entertainment districts.

Farmer and Clifford (2021) undertook a mapping review of patron banning policy and practice across Australian jurisdictions and identified a common theme which supports their underlying rationale. It is assumed that the risk of exclusion from licensed venues or public areas will address immediate issues arising from alcohol-related disorder, and act as a deterrent to manage the future risk of problematic behaviours. This expectation is discussed in depth by Farmer, Curtis and Miller (2018) in relation to the proliferation of police-imposed banning provisions across Australian jurisdictions. Bans which remove individuals from a specific place can provide an immediate response to a behavioural issue within a given location, and may prevent a recurrence if the ban is enforced in a proactive and effective manner. However, the specific effects on patron behaviours and any broader deterrent effects are less clear.

In Australia, there is limited research examining the effects of any banning provision, with particular regard to individual or community perceptions, compliance or subsequent behavioural decision making. Farmer (2021a, b) interviewed recipients of licensee barring orders imposed in the Australian state of Victoria and found minimal evidence of their capacity to act as a tangible deterrent or effective agent for behaviour change. Curtis et al. (2018) explored public understanding of patron banning in Victoria via an online questionnaire. The majority of respondents demonstrated little knowledge, and Curtis et al. questioned the extent to which banning fulfils the expectations of a general deterrent. Curtis et al. (2022) examined the offending records of a small sample of banning notice recipients from one location in Victoria, and found no demonstrable reduction in the quantum or severity of offending behaviours following receipt of a ban. Interview research conducted in Victoria and the Australian state of Queensland (Miller et al. 2016, 2019) examined the perceived effects of patron banning and found support for banning as a deterrent to anti-social behaviours. However, neither study engaged directly with ban recipients to explore their response to the ban, nor explored broader community awareness.

The focus of this paper is the state of Western Australia (WA) where, in addition to licensee bans, police-imposed barring notices and prohibition orders primarily target alcohol-related violence and disorderly behaviours. Each provision is presumed to prevent crime, and increase public safety, by deterring the recipient from further behaviours which may lead to another ban; and deterring the wider community from engaging in behaviours which may lead to a ban.

#### Venue-Specific Licensee-Imposed Bans

Under WA’s *Liquor Control Act* 1988 (s.115), a licensee or a police officer may refuse entry to a licensed venue or issue an immediate exclusion for a specified period for reasons that include appearing to be drunk or behaving in an offensive manner. Failure to comply can result in a fine of up to AU$5,000.

#### Police-Imposed Barring Notices

Section 115AA of WA’s *Liquor Control Act* 1988 empowers the Commissioner of Police, or a police officer above the rank of Inspector, to approve the exclusion of an individual from a specified licensed venue, or a class of licensed venues, if there is reasonable belief that the recipient has “(a) been violent or disorderly; or (b) engaged in indecent behaviour; or (c) contravened a provision of any written law” (*Liquor Control Act* 1988, s.115AA). The original legislation, passed in 2010, required the alleged behaviour to have occurred within a licensed venue. A 2018 amendment expanded the permissible imposition of barring notices to cover the vicinity of licensed premises (*Liquor Control Act* 1988, s115AA(2)) to include persons misbehaving in queues, on footpaths or in car parks close to licensed premises. There is no specific distance which denotes the ‘vicinity’ of a licensed venue.

Unlike most other Australian jurisdictions, police-imposed barring notices in WA are not issued on-the-spot (Farmer & Clifford 2021; Farmer, Curtis & Miller 2018). Incidents are reported to a centralized Liquor Enforcement Unit (LEU), and a determination is made on the balance of probabilities following consideration of evidence, such as CCTV and witness statements. A barring notice can be imposed for any period up to 12 months, and the penalty for breaching the notice is a fine of up to AU$10,000 (*Liquor Control Act* 1988, s.115AA).

#### Department of Racing, Gaming and Liquor (RGL)-Imposed Prohibition Orders

Following the passage of the *Liquor and Gaming Legislation Amendment Act* 2006, section 152 of WA’s *Liquor Control Act* 1988 legislated for the imposition of prohibition orders as a response to more serious anti-social, disorderly and violent behaviours. The Commissioner of Police may seek approval from the Director of Liquor Licensing to prohibit an individual from “... entering specified licensed premises, licensed premises of a specified class or any licensed premises” (*Liquor Control Act* 1988, s.152B). The scope of incidents which may meet the criteria for an application is wider than that for a barring notice. Prohibition orders can be issued to people who are involved in more serious alcohol-related anti-social behaviour, or who have been convicted of relevant offences and for whom exclusion from licensed venues is regarded as necessary. There is no requirement for the behaviours or offences to have occurred in or around licensed premises. A prohibition order can be issued on the balance of probabilities, and imposed for up to five years for an adult and two years for a juvenile (*Liquor Control Act* 1988, s.152F). An alleged breach or failure to comply with an order can result in a fine of up to AU$10,000 (s.152L).

## Research Method

The wider study from which this paper is drawn was initiated by WA Police, in collaboration with Deakin University, to examine the effect/s and effectiveness of barring notices and prohibition orders. The existence of patron banning provisions and the knowledge of their effect/s are presumed to act as a deterrent to prevent unacceptable behaviours by all patrons. For example, the parliamentary debates which preceded the introduction of barring notices in WA emphasise the expectation of their general deterrent effect (WA Parliamentary Debates 2010). Other than a limited study by Curtis et al. (2018), there is little understanding of how much is known about the banning mechanisms that can be used within Australian jurisdictions, or the extent to which knowledge of patron banning may affect the behaviours of the general population. This paper examines the level and extent of public awareness of the provisions as an essential pre-requisite for general deterrence. It reports relevant findings from two parts of the study: a panel survey and stakeholder interviews.

### Procedure

#### Panel Survey

A panel survey was undertaken to explore community knowledge and perceptions of the banning provisions currently in operation in WA. The survey was co-ordinated by Qualtrics and the sample was drawn from across WA. The survey sought to focus on members of the community who were more likely to be regular or occasional visitors to entertainment precincts, and for whom patron banning is intended to be a deterrent. It is acknowledged that, even among this cohort, not everybody will need to be deterred from undesirable behaviours, but it is this cohort for which awareness of patron banning is most likely to influence its potential general deterrent effect. Qualtrics used a sampling algorithm to ensure that the majority of respondents attended licensed venues at least weekly or monthly. The sampling also targeted participants aged between 18 and 45 years — as data provided by WA Police confirmed that approximately 90% of barring notice and prohibition order recipients fall into this age category - but did not exclude people older than 45. The survey included key demographics of respondents but no identifiable respondent details were recorded.

The panel survey examined awareness and knowledge of patron banning in WA and gathered the opinions of respondents regarding its effect and effectiveness. The scope included licensee banning as well as police-imposed barring notices and prohibition orders. The survey attempted to discern any differences in understanding and perceived effect. Survey questions covered four key domains: (1) respondent knowledge of police barring notices and prohibition orders; (2) from where respondents obtained their knowledge: for example, direct experience, social media; (3) Perceived awareness of patron banning among respondent peers; and (4) perceptions and perceived effectiveness of patron banning.

#### Stakeholder Interviews

Stakeholder interviews were conducted to develop a picture of experiences and perceptions of patron banning in WA. Interviews have provided a particularly informative element of previously successful projects in this area (e.g. Miller et al., 2012 (DANTE); 2019 (QUANTEM); 2022 (LEARNT)). Stakeholders comprised three key categories.

#### Venue Owners/Managers/Licensees

The contact details of on-premise (bars, pubs etc.) liquor retailers across WA were sourced from online listings, with care taken to include venues of different types and in a range of locations. In total, 145 venues were contacted, of which 88 generated no response, 6 declined and 51 expressed interest. In addition, the coordinators of four WA Liquor Accords (in Perth, Fremantle, Mandurah and Scarborough) forwarded project details to their member lists.

#### WA Police

WA Police facilitated contact with current and previous members of the Liquor Enforcement Unit, all of whom were invited to participate.

#### Other Key Informants

A range of other stakeholders were drawn from ID scanner companies; peak bodies (an Australian term for advocacy groups or trade associations) covering on-premise and packaged liquor sales; the Department of Racing, Gaming and Liquor (RGL); Registered Training Organisations (RTOs) which deliver RGL Approved Manager training (mandatory accreditation for licensees in WA); and Liquor Accord co-ordinators. All were contacted directly using publicly available information and invited to participate.

For each cohort, those who expressed interest were sent a Plain Language Statement (PLS), which provided an overview of the project, and a consent form. Project researchers then followed up to schedule interviews with those who wished to participate. Interviews were conducted online or by telephone, other than one participant who preferred to provide a detailed written response. The effects of the COVID-19 lockdowns and restrictions, as well as the more usual demands of running a venue, meant that not all of those who expressed interest were interviewed.

The interviews were semi-structured and questions were presented across four domains: (1) Awareness/experiences of and attitudes to police barring notices and prohibition orders; (2) data and information flow; (3) knowledge and understanding; and (4) current issues (e.g. barriers to implementation/enforcement of patron banning provisions; perceptions of impact; deterrent effects; recommendations for improvement; broader or other specific challenges). Any emergent themes or issues brought up by the interview questions were also explored.

#### Ethical Approval

Deakin University ethical approval was obtained in November 2020 (reference: HAE-20-163). Approval was also received from the WA Police Research Governance Unit as part of the overall project approval.

#### Analysis

The panel survey was implemented by Qualtrics in March 2021, with a final dataset comprising 1018 valid responses. Qualtrics removed responses that contained erroneous non alpha-numeric data or were clearly non-sensical. The dataset was examined using a range of analytical tools within the Qualtrics platform. Cross-tabulations were used to establish the significance of associations between responses and/or their respondents. Inferential tests of significance, such as chi-squared, *t*-tests and ranked ANOVA, were applied according to the specifics of each sample.

Between April and June 2021, interviews were conducted with 54 stakeholders. Interviews were transcribed and returned to the participants for review, after which each transcript was de-identified and a pseudonym assigned. Transcripts were analysed using thematic analysis, an inductive reflexive design where, rather than approach a problem with a theory already in place, the researcher identifies and explores common themes which arise within the data being examined (Braun & Clarke, 2006; Kellehear, 1993). Where available, narratives offering opposing viewpoints were included (Pope & Mays, 1995). As the questions developed for the project formed the basis of the initial themes, it was appropriate to undertake a template thematic approach (Brooks & King, 2014). The analysis followed six key steps: (1) data familiarity, (2) preliminary coding, (2) organisation of themes into meaningful clusters, (4) define an initial template for coding, (5) apply the initial template to further data, and modify where needed and (6) finalise the template to apply it to the full data set (Brooks & King, 2014). The interviews were not designed to give definitive answers, but they constitute a rich body of qualitative data that provides important ‘on the ground’ perceptions and context. Opinions were diverse, but common themes emerged. Example quotations are included within the “Results” section to illustrate the findings of relevance to this paper.

### Study Limitations

It is acknowledged that the survey sample and their views, experiences, knowledge and awareness are not necessarily representative of the wider community, or of the population whose awareness of the banning provisions is most likely to benefit the community or themselves. Most respondents did not report ever receiving any type of patron ban, but this does not undermine the core purpose of the survey — which was to discern the level of public awareness and understanding of the provisions among the population most likely to be affected by patron banning. Additional research is required to examine ban recipient perceptions and experiences in more depth.

For the interview component of the study, the method of recruitment for licensees/venue managers limits the representativeness of the sample. While it is gratifying that 24% of the venues which were ‘cold’ contacted agreed to participate in the study,^1^ it is acknowledged that 76% of the venues contacted did not take part. Despite the effects of patron behaviours for venues and their staff, the majority chose not to participate when invited. Across all stakeholder types, 54 interviews were completed. Where possible, all stakeholders within a category (e.g. RTOs, LEU officers) were contacted and invited to participate. However, as participation was voluntary the project could only interview those who accepted the invitation. It is possible that those who agreed to participate did so because of particular experiences and/or strongly held views which may not be representative of their cohort.

The effects of COVID-19 upon freedom to move across Australia affected the original intention to initiate and undertake the stakeholder interviews in person. As a result, most of the interviews took place online (via Zoom or MS Teams) or by telephone. Venue manager availability was variable during and following periods of lockdown. However, neither issue notably affected the number of interviews completed, the quality of the interactions or the information that was discerned.

## Results

### Panel Survey

The 1018 survey respondents were located across 157 different WA postcode areas. Respondents from in/around the state capital of Perth comprised 47% of the sample. Most of the respondents (75%) were aged between 18 and 35 years; 22% were aged between 36 and 45 years; and 2% were over 45. Most of the respondents (79%) attend licensed premises on a regular basis (weekly or monthly).

#### Perception of Patron Banning

Table 2 summarises the perceived effect and effectiveness of patron banning in general. Most respondents (66%; *n*=674) perceived patron banning to be moderately or extremely effective. Displacement was the most commonly perceived effect of patron banning, noted by 59% of the 1018 respondents (*n* = 599). Only 19% (*n* = 192) perceived that a banned person would change their behaviours/reduce future offending, and 10% (*n* = 108) believed that there would be no effect on recipient behaviour.

**Table 2:** Survey respondents: perception of banning effectiveness and effect/s (*n*=1018)

Effectiveness of patron banning	Number	Percentage
Extremely	168	17%
Moderately	506	50%
Slightly	274	27%
Not at all	70	7%
Most likely effect of a ban		
Displacement	599	59%
Pride/badge of honour	101	10%
Reduce offending	192	19%
Nothing	108	11%
Other	18	2%

#### Self-Reported Ban Recipients: Limited Effect on Offending

A total of 35 of the 1018 respondents (3.4%) self-reported as recipients of one or more of any type of patron ban. This is summarised in Table 3. Respondents who had received more than one ban were asked to address follow-up questions with respect to their first ban.

**Table 3:** Survey respondents: self-reported ban recipients (*n*=35)

Number of Bans	Number	Percentage	
One	23	66%	
Two	7	20%	
Three +	5	14%	
Type of ban	Number	Percentage	
Licensee	27	77%	
Barring notice	2	6%	
Prohibition order	4	11%	
Not sure	2	6%	
Length of ban	Number	Percentage	
Up to 1 month	17	49%	
1–3 months	7	20%	
3–6 months	3	9%	
6 months +	8	23%	
Ban deserved	Number	Percentage	
Yes	12	34%	
No	10	29%	
Maybe	13	37%	
Contested ban	Number	Percentage	Successful
Yes	12	34%	0 (0%)
No	23	66%	
Charged with offence before first ban	Number	Percentage	For action in/near licensed premises?
Yes	13	37%	11 (85%)
No	22	63%	
Charged with offence since first ban	Number	Percentage	For action in/near licensed premises?
Yes	10	29%	8 (80%)
No	25	71%	

Respondents who had been banned were significantly more likely to report that they attend pubs/bars on a regular basis (*p*<0.001) and more likely to be male (*p*<0.05). The majority reported that their ban was or may have been deserved (71%, *n*=25), but there was no significant effect identified with respect to subsequent offending. While the sample is small, 29% (*n*=10) reported being charged with another offence after receiving their ban, compared with 37% (*n*=13) who reported being charged with an offence before their ban. For both cohorts, most charges related to behaviours in or near licensed premises.

#### Limited Awareness and Understanding of Patron Banning Provisions

The overall level of awareness and understanding of barring notices and prohibition orders was assessed by asking respondents to first indicate their awareness of each provision. Those who answered ‘yes’ were asked to provide a brief explanation of the provision. Responses were placed into one of four categories (with the data analysed and categorised by a single researcher): *no understanding*: the respondent either left the field blank or noted that they were not able to explain the provisions; *incorrect understanding***:** the explanation provided was incorrect. *Basic understanding:* the explanation provided was broadly correct but was simplified and/or missed key details; *clear understanding*: the explanation provided demonstrated a solid awareness of the provision including specific details, such as possible reasons for imposition, who can impose it and/or awareness of the permitted lengths. Table 4 indicates the number and proportion of respondents who confirmed awareness of each provision. Of those respondents who answered ‘yes’, the accuracy of their demonstrated understanding is noted, along with the self-reported source/s of their awareness.

**Table 4:** Survey respondents: awareness and understanding of barring notices and prohibition orders (*n*=1018)

Awareness of Barring Notices	Number	Percentage of 1018	
No	764	75%	
Yes	254	25%	
Of those who indicated ‘yes’	Number	Percentage of 254	Percentage of 1018
Awareness from			
Hearsay/conversation	71	28%	
Experience (own/other)	61	24%	
News media	73	29%	
Other/not Sure	49	19%	
Demonstrated understanding			
None stated	60	24%	6%
Incorrect	81	32%	8%
Basic	92	36%	9%
Clear	21	8%	2%
Awareness of prohibition orders	Number	Percentage of 1018	
No	889	87%	
Yes	129	13%	
Of those who indicated ‘yes’		Percentage of 129	Percentage of 1018
Awareness From			
Hearsay/conversation	32	25%	
Experience (own/other)	30	23%	
News media	38	30%	
Other/not Sure	29	22%	
Demonstrated understanding			
None stated	33	26%	3%
Incorrect	70	54%	7%
Basic	24	19%	2%
Clear	2	2%	0.2%

A quarter of respondents acknowledged awareness of barring notices (*n*=254), with 13% aware of prohibition orders (*n*=129). Respondents who reported more frequent attendance at pubs/bars were more likely to be aware of the barring notice provisions (*p*<0.01). There were no relevant associations with respect to prohibition orders. Overall, 89% of the survey respondents demonstrated no understanding of barring notices, and 97.5% demonstrated no understanding of prohibition orders.

For respondents who indicated awareness of either provision, hearsay, personal experience and news media were consistent sources of their knowledge.

#### Awareness of Provisions Higher with Direct Experience

Table 5 summarises the findings with respect to respondent experience and first-hand awareness of patron banning: 24% of the 1018 respondents (*n*=247) reported knowing somebody who has received any type of patron ban in WA. These respondents were significantly more likely to be aware of licensee banning provisions (*p*<0.001), and more likely to be aware of police barring notices (*p*<0.01) and prohibition orders (*p*<0.01). Respondents who had received a patron ban themselves were significantly more likely to be aware of licensee banning provisions (*p*<0.001), and more likely to be aware of police barring notices (*p*<0.01) and prohibition orders (*p*<0.05).

**Table 5:** Survey respondents: banning experience and awareness of acquaintances (*n*=1018)

Know recipient of a patron ban	Number	Percentage	
No	771	76%	
Yes	247	24%	Percentage of ‘Yes’ Responses
Licensee	198		80%
Barring notice	79		32%
Prohibition order	46		19%
Friends aware of patron banning	Number	Percentage	
Definitely yes	79	8%	
Possibly yes	571	56%	
Definitely no	215	21%	
Not sure	153	15%	

### Interviews

Table 6 summarises the number and percentage of interview participants by stakeholder type. Venue participants comprised the majority of the sample (65%) and were drawn from across WA: 28 of the venues (80%) were located in the Greater Perth area (including Perth, Fremantle, Scarborough), where the key entertainment districts are found. The remaining venues (20%) were located elsewhere in WA (including the more remote Kimberley and Gascoyne regions).

**Table 6:** Summary of stakeholder participants

Stakeholders	*N*	Percentage of total*	Pseudonym code
WA Police	9	17%	LEU
Venue owners/managers/licensees	35	65%	Venue
Other Key Informants (RGL, RTOs, liquor accords, peak bodies, ID scanner companies)	10	19%	KIP
Total	**54**		

^*^May not add up to 100% due to rounding

The interviews examined perceived public awareness and key informant perspectives regarding the wider deterrent effect/s of barring notices and prohibition orders. It is acknowledged that some interviewees may have responded with respect to patron banning more generally.

Supporting the findings from the panel survey, the perceived level of public awareness of barring notices and prohibition orders was limited. This perception was particularly clear and consistent among the venue and police participants. Interviewees reported a general absence of information and education about patron banning, which translated to a belief that most people have no knowledge of provisions that are available or the consequences for recipients. The key exceptions were believed to be patrons who had received a ban or had direct contact with the provisions. One LEU officer stated: “I don’t think the general community know. If you don't get into trouble, you're not aware of anything that happens outside your little bubble” [LEU-04]. The same perspective was evident from most venue participants:

As in general public awareness? … I think the general public, probably most of them, have no idea whatsoever. [Venue-06]

I don't think there's much awareness of it at all. I think…the only people who are really aware of it are people that get banned. [Venue-15]

Stakeholders perceived the level of public awareness to be greater within smaller and more regional communities. The rationale being that people in these areas are more aware of individual behaviours and specific events, whereas larger towns and urban centres tend to be busier with less focus on individuals and more potential for anonymity:

I think in regional areas, people would probably be a bit more aware of them, because in those areas I think they’d be a lot more effective… but in cities I doubt anyone outside of people working in the industry would be aware that they even really exist. [Venue-27]

The stakeholders identified limited access to information as a key reason for the lack of awareness of the provisions. One venue participant noted:

… they’re not very well known unless you're in the circle. Unless you've got the information in the background … There's nothing really written anywhere about them unless you have physically gone and asked someone about it…[Venue-34]

Crucially, stakeholders directly linked higher levels of public education and awareness of the provisions with a stronger potential deterrent effect. This perspective is exemplified by these three observations:

I think if there was more of a I guess a community advertising campaign to make people aware of it, it would work better as a deterrent… [KIP-01]

Some do change their ways, but where it’s effective isn’t in the people that have been banned changing their ways. Where it’s effective is that people know not to kick off because they don’t want to be banned. So, it's more effective on the people who might do the wrong thing than the people that have done the wrong thing… It’s the fear of it. At the moment these kids are coming out of high school with no fear…There’s no consequence for these kids. They have to know that coming into a venue isn’t a right, it's an absolute privilege. [Venue-03]

I think if people knew about it, it definitely would be [a deterrent] [Venue-20]

To address the perceived lack of awareness of the provisions, a common recommendation, particularly among venue participants, was to improve public awareness of behavioural expectations in and/or around licensed venues. Suggestions included focused education campaigns, both on premise, by way of signage, and more generally across the community:

Certainly, I reckon you could spell it out to them on one of those sort of signs … I don't know how the wording would go... [Venue-09]

… education towards the public of what they can and cannot do in a licensed premise. I think it's important. As I said before, I think a lot of people don't realize if they're told to leave, they have to leave. It's not about standing and arguing. That is the rule … but the public needs to be aware of that… [Venue-32]

Oh, I just think something needs to go out there every now and then. Even just an ad. Just throw something out there every now and then just to say ‘look, if you act up, these things can happen’. [So, you're thinking television advertisement?]. Anything will do. Even just posters. If we had something sent out to each venue as a poster and put up on a wall and you'd probably create knowledge. [Venue-34]

## Discussion

Patron banning provisions are intended to address anti-social and disorderly behaviours with a rationale that encompasses specific and general deterrence. For such provisions to have a general deterrent effect, this requires a reasonable level of public knowledge and understanding. Unlike much of the research that has examined the effects and effectiveness of comparable provisions in other jurisdictions, such as ASBOs and dispersal orders (Burney 2002, 2009; Crawford, 2009a, b), this paper explores the level of public knowledge and understanding of barring notices and prohibition orders, as part of a study analysing the wider deterrent and other effects of both provisions. It is important to emphasise that this paper has not sought to establish the actual general deterrent effect of patron banning in WA or elsewhere.

It is reasonable to wonder why those who may never behave in an anti-social manner need to be aware of provisions that can be imposed on those who do. In any social group or situation, any individual may react or behave inappropriately in response to a range of factors, and others may not. Individual awareness of possible consequences can influence personal behaviours, but responses to risks are individualised, with some people more likely to be deterred than others (Jacobs 2010; Matthews & Agnew 2008; McGrath 2009). As the rationale for the barring notices and prohibition orders presumes a general deterrent effect, it is reasonable to explore the extent to which this may be present. The first step in doing this is to explore public awareness. As it is not possible to identify every person who does need to be deterred, this study focused on survey respondents who are more likely to attend licensed premises. The potential role played by general awareness and understanding is of particular relevance for this cohort. The anti-social behaviours for which patron bans, ASBOs and other provisions are typically imposed reflect both individual and community contexts. For patron bans, the latter is more prevalent as most are imposed following behaviours occurring in public places and/or where other people gather (e.g. pubs, bars and other licensed venues). Greater public awareness of the potential use of patron bans may help to forestall the potential escalation of inappropriate behaviours. This may seem aspirational, but this expectation is evident within the underpinning rationale for patron bans that they will reduce crime and increase public safety (Farmer, Curtis & Miller, 2018).

The panel survey revealed a low level of community understanding and awareness of police-imposed barring notices and prohibition order provisions: 75% of respondents had not heard of police barring notices, and 89% had no demonstrated understanding; 87% of respondents had not heard of prohibition orders, and 97.5% had no demonstrated understanding. These findings undermine expectations of the broader deterrent effects of patron banning. For the majority of respondents, where knowledge has not been derived from direct experience, the primary inputs were general conversations or news/media, both of which have a clear potential for misinformation. This risk is demonstrated, to some extent, by the findings of the survey: as the level of public awareness of patron banning that was perceived by respondents was found to be higher than demonstrable knowledge. The interviewees were consistent in their perception of a low level of awareness and understanding of the patron banning provisions, particularly among the police participants and those connected with licensed premises. Most interviewees reported that, unless directly affected, most patrons had no awareness of any banning measures. These perspectives support, and are supported by, the panel survey findings.

An effective policy encompassing an expected community deterrent requires not only public knowledge of the provisions, but also public awareness of their intended effect. Although demonstrable awareness of each of the WA provisions was limited, the panel survey respondents provided general support for the principle of patron banning, with 66% believing banning to be moderately or extremely effective. Concerns were expressed about the possibility of displacement - banned patrons being able to go elsewhere and, as a result, not having any incentive to change their behaviour. This was raised as an issue in other Australian jurisdictions (Curtis et al. 2022; Taylor et al. 2021) and by Sogaard (2018) in relation to zonal banning in Denmark, and can serve to limit the specific and wider deterrent effects of bans.

The generally positive perception of the effectiveness of patron banning in WA provides a strong starting point. Better understanding may act to further strengthen the perceived value of the provisions as well as potentially embedding and extending their general deterrent effect. In addition to providing a clear, and in places immediate, response to anti-social or disorderly behaviour in and around licensed venues, patron banning policy is based upon a presumption that the experience of being banned will have a deterrent effect: to encourage those affected to not engage in problematic behaviours in future, and to deter others from similar behaviours for fear of being banned. The latter, a broader, community based deterrent effect, requires the community to be aware of the provision that, through good behaviour, they are seeking to avoid. The findings of this study suggest that this aspect of banning policy in WA would benefit from a number of operational refinements. The policy implications addressed here draw from other Australian jurisdictions and focus primarily on Western Australia, given the way in which the patron banning provisions are imposed and enforced there. However, the broader implications are relevant to comparable provisions in any jurisdiction — where benefits may be derived from changes which enhance and ensure broader public awareness, and support expectations of general deterrence.

The successful implementation of any policy that excludes recipients from defined locations requires timely, accurate and appropriate enforcement. A key criticism of ASBOs in England and Wales reflected a perception of overly punitive imposition and enforcement, evidenced by high breach rates (Squires & Stephen 2005). Crawford and Lister (2007) pointed to better communication as a way to increase knowledge and the potential beneficial effects of dispersal orders. In Australia, police officers are primarily responsible for the ‘on the ground’ enforcement of banning provisions but this relies on the support of licensees, venue managers and their security personnel — to identify banned patrons and, where appropriate, alert police to potential breaches of bans. Typically, such ‘third party policing’ (Mazerolle & Ransley, 2002) of patron bans requires access to photographs and other personal details in order to identify banned patrons, but technological solutions can help with enforcement (de Andrade, Coomber, et al., 2021a, b; Miller et al., 2012; 2016; Palmer, Warren & Miller, 2011; 2014). ID scanners can check the identity of people who attempt to gain entry to a venue and to detect patrons who have been banned. The scanned patron ID documents (such as a driver’s licence or passport) can be matched against a database of banned patrons.

WA does not currently operate a mandated ID scanner network across all venues. Details of banned persons are made available to licensees manually (typically via an online portal), and pre-emptive and proactive monitoring of banned patrons is difficult. Enforcement challenges were identified as a key concern across the stakeholder interviews, reflecting limited and cumbersome information flows and the reliance on manual identification of banned patrons (this is addressed in a separate paper, currently under review). More effective enforcement, through the wider use of visible methods such as ID scanners, may serve to increase awareness of barring notices and prohibition orders among patrons and, as a result, may enhance the wider deterrent effect of these banning provisions. In July 2017, as part of the Australian state of Queensland’s Tackling Alcohol Fuelled Violence (TAFV) Policy (Miller et al. 2019), ID scanners were made mandatory within late trading licensed venues (those trading after midnight) located in specific entertainment precincts. This ID scanner implementation has been associated with reductions in police-recorded assaults (Coomber et al., 2021), ambulance attendances (Coomber et al., 2021; de Andrade, Coomber, et al., 2021a, 2021b), and improved outcomes with respect to the resolution of alleged crimes by the Queensland Police Service (Coomber et al., 2021; de Andrade, Puljević, et al., 2021a, b). However, it is acknowledged that it can be difficult to discern the particular effects of individual provisions that are implemented as part of a broader policy initiative, such as the TAFV policy.

To strengthen the potential deterrent effect of the banning provisions in WA, the interviewees highlighted the value of better communication and more effective information sharing in order to increase the level of community awareness. Suggestions included the wider use of high impact signage within licensed premises, more effective use of social media to raise awareness of patron banning policy, perhaps by publicising newsworthy cases within a local area, and the expansion of school and community education programs. While not universally supported, the better use of signage in and around venues is one option. In Victoria, for example, there is a range of required signage to be used within licensed premises (VCGLR, 2022). All signs are available online and can be downloaded and printed at any time. Failure to display the required signage carries a financial penalty under Section 102 of the *Liquor Control Reform Act 1998*. In the Victorian cities of Geelong and Shepparton, the ‘do you know’ education campaign positioned posters on the door of every venue, setting out the fines for anti-social behaviours (Greater Shepparton City Council, nd).^2^ Signage presents a low-cost operational change that could be implemented immediately, if initially on an optional basis. To optimise the effect/s of banning policy, the WA Government should work with WA Police, the RGL and peak bodies such as the Australian Hotels Association - Western Australia and the Liquor Stores Association to identify how to further expand community awareness and understanding of the patron banning provisions, as well as behavioural expectations more broadly.

## Conclusion

The panel survey and stakeholder interviews both confirm a generally low level of public awareness and demonstrated understanding of the patron banning provisions that are currently operational in WA. As may be expected, awareness was higher for those people who have been directly affected or who know someone who has been directly affected. It is difficult to account for other influences on general deterrence beyond knowledge of the provisions. But any policy that can lead to a restrictive penalty relies, for its effectiveness, on sufficient knowledge of that restriction and reasonable fear of it being imposed and enforced. It is, therefore, essential to ensure that the patron banning provisions currently operational in WA are seen to be enforced proactively, that their use is publicised widely, and that they are monitored and scrutinised to continue to optimise their effect/s and effectiveness. Patron banning policies in other jurisdictions may benefit from similar refinements.

## Footnotes

1 By way of comparison, the QUANTEM study in Queensland reported that 8.2% of key informants who were ‘cold’ contacted completed an interview (Miller et al. 2019, p.532).

2 An example sign can be accessed via this link: https://greatershepparton.com.au/assets/files/documents/community/health_safety/So_You_Know_Venues.pdf
